# Application of Sini Decoction at acupoint on gastrointestinal dysfunction in patients with sepsis: A clinical study

**DOI:** 10.1097/MD.0000000000040464

**Published:** 2024-11-01

**Authors:** Yuteng Du, Jingjing Hu, Pingping Zhang, Ting’ai Ge, Yidan Zhou

**Affiliations:** a Department of Emergency Medicine, Hangzhou Third People’s Hospital, Hangzhou, Zhejiang Province, PR China.

**Keywords:** gastrointestinal dysfunction, interleukin-6, lactate, motilin, sepsis, Sini Decoction

## Abstract

The occurrence of gastrointestinal dysfunction is widely recognized as a prevalent complication in patients with sepsis. To investigate clinical effect of Sini Decoction at acupoint on gastrointestinal dysfunction in sepsis patients. Seventy-five patients with gastrointestinal dysfunction caused by sepsis were randomly divided into 2 groups. Treatment group received routine Western medicine treatment combined with Sini Decoction at acupoint, while control group treated with talcum powder at acupoint. Treatments in both groups lasted 7 days. Changes in the acute physiology and chronic health evaluation II score, sequential organ failure assessment score, mechanical ventilation duration, the length of Intensive Care Unit (ICU) stay, enteral nutrition tolerance scores, abdominal circumference, gastric residual volume, bowel sounds, and serum index were observed. After treatment, the enteral nutrition tolerance score, abdominal circumference, gastric residual volume, and levels of lactate and interleukin-6 were significantly lower in the treatment group compared to the control group. Bowel sounds were more active and motilin levels were higher in the treatment group. Additionally, the length of ICU stay was significantly shorter in the treatment group than in the control group. Our findings demonstrated that the application of Sini Decoction at acupoints in sepsis patients with gastrointestinal dysfunction can effectively enhance gastrointestinal function, leading to a reduction in ICU stay duration and an improvement in patients’ prognosis.

## 1. Introduction

The prevalence of sepsis imposes a significant burden on the global healthcare industry and contributes to the leading cause of mortality in Intensive Care Unit (ICU). The substantial incidence and mortality rates associated with sepsis have undeniably placed a considerable strain on the global health system.^[1–3]^ The incidence of this severe systemic infection is not only high, but it also presents a grave condition with an exceptionally high fatality rate, posing a significant threat to human health. The management of sepsis incurs substantial costs and consumes extensive medical resources. The aforementioned statement highlights the urgency of a collective global health endeavor, thereby emphasizing its status as a pressing worldwide health predicament.^[4]^ Sepsis, as a severe systemic infection, entails a multifaceted pathogenesis characterized by the dysfunction of multiple organ systems. Gastrointestinal dysfunction is among the prevalent complications observed in sepsis patients, significantly impacting nutritional absorption, immune function, and disease prognosis.^[5]^ The exploration of the mechanism underlying gastrointestinal dysfunction caused by sepsis and the development of intervention strategies hold significant implications for enhancing the efficacy of sepsis treatment and improving patients’ quality of life. Longhitano Y, et al have discovered that gastrointestinal mucosal injury, disorder of intestinal microbial flora, and impaired intestinal motility are the most prevalent manifestations of gastrointestinal dysfunction in septic patients. Consequently, investigating methods to promptly enhance gastrointestinal function and restore the integrity of the gastrointestinal barrier has become a crucial focus in sepsis prevention and treatment research.^[6]^

Dietary intake plays a crucial role in the management of sepsis. Adequate nutritional support can enhance patients’ immune response, bolster their resistance against pathogens, and facilitate their recovery.^[7]^ The available evidence suggests that daily supplementation of vitamins and trace elements through diet may effectively support the maintenance of immune system function in patients with sepsis. The dynamic changes in Zinc (Zn) levels associated with the severity of sepsis can be utilized for assessing sepsis severity and predicting the prognosis of sepsis biomarkers. The antioxidant function of Vitamin C is demonstrated through its scavenging of free radicals. In animal models of sepsis, the exogenous administration of Vitamin C has been shown to enhance capillary perfusion, increase the responsiveness of arterioles to vasoactive drugs, and improve organ function.^[8–11]^ In certain sepsis patients, a deficiency of Vitamin B1 has been observed, prompting the suggestion that the administration of Vitamin B1 may contribute to ameliorating the metabolic state of these patients.

The treatments for sepsis mentioned by Evans L, et al in the *Surviving Sepsis Campaign: International Guidelines for Management of Sepsis and Septic Shock 2021* encompass antibiotics, fluid management, vasoactive agents, and additional therapies.^[12]^ Traditional Chinese medicine (TCM), rooted in the theory of Yin and Yang 5 elements, stands as one of the most ancient medical doctrines. The establishment of TCM’s theoretical system has undergone extensive exploration and accumulation, representing the crystallization of wisdom from ancient physicians. *The Inner Canon of Huangdi* and *Compendium of Materia Medica*, renowned classics in TCM, have laid a robust foundation for the advancement of this field. In addition to conventional Western medical treatments, clinical observations in China have revealed that TCM also demonstrates a certain efficacy in ameliorating gastrointestinal dysfunction induced by sepsis, encompassing techniques such as acupoint stimulation, herbal enemas, and acupuncture.^[13]^ Hereby, we conducted a preliminary investigation to collect and analyze clinical data from sepsis patients with gastrointestinal dysfunction who were treated with Sini Decoction at acupoints combined with live bifidobacterium, lactobacillus, and enterococcus powder. We compared these patients with those who received talcum powder at acupoints combined with live bifidobacterium, lactobacillus, and enterococcus powder. The possible underlying mechanism was thoroughly discussed, aiming to propose a novel treatment strategy for patients with sepsis-induced gastrointestinal dysfunction.

## 2. Methods

### 2.1. Study design and participants

The aim of this retrospective study was to analyze the application of Sini Decoction at acupoints in patients with sepsis and gastrointestinal dysfunction, comparing it with talcum powder treatment at acupoints. Electronic medical records of sepsis patients treated at the Department of Emergency Intensive Care Unit, Hangzhou Third People’s Hospital from June 2022 to January 2024 were reviewed and evaluated. According to a random number table, we recruited 60 patients for our study with a 2-tailed α = 0.05 and power of 75%. Taking into consideration an anticipated compliance rate of 80%, we invited a total of 75 patients to participate in our study. These participants were then randomly assigned to 2 groups: the treatment group consisting of 38 patients and the control group consisting of 37 patients. The subjects of this study consisted of sepsis patients with gastrointestinal dysfunction who were treated with Sini Decoction at acupoints, while the control groups included patients treated with talcum powder at acupoints.

The inclusion criteria of sepsis is based on *Surviving Sepsis Campaign: International Guidelines for Management of Sepsis and Septic Shock 2021,* with a minimum age requirement of 18 years or older.^[12]^ The assessment of gastrointestinal dysfunction in patients is based on *Gastrointestinal function in intensive care patients: terminology, definitions and management. Recommendations of the ESICM Working Group on Abdominal Problems.*^[14]^ Exclusions apply to patients undergoing digestive system surgery, experiencing gastrointestinal dysfunction caused by primary gastrointestinal disease, diagnosed with digestive system tumors, pregnant women, patients who pass away within 48 hours of admission to the ICU, and those participating in clinical trials for other medications. The study received approval from the Ethics Committee of Hangzhou Third People’s Hospital (No. 2022KA021).

The study was approved by the Ethics Committee of Hangzhou Third People’s Hospital (No. 2022KA021). The studies were conducted in accordance with local legislation and institutional requirements. Written informed consent was obtained from each patient or their legal representatives.

### 2.2. Treatment

Patients were divided into 2 groups as treatment group and control group, and given anti-infection, bifidobacterium, lactobacillus and enteroccus powder (Shanghai Shangyao Xinyi Pharmaceutical Factory Co., Ltd) and basic organ support treatment.

Each group received the same food intake. Enteral nutrition was initiated within 24 hours of enrollment, with a target caloric requirement set at 20 to 25 kcal/kg per day. Additionally, the protein need goal was established as 1.2 to 2.0 g/kg/d, while the calorie/nitrogen ratio was determined to be in the range of 100 to 200:1. The administration of parenteral nutrition was initiated when enteral nutrition failed to achieve more than 60% of the desired goal. Glucose accounted for 50% to 75% of the total caloric requirements, while the calorie-to-lipid ratio was established at 1 to 3:1. Additionally, adequate provision of electrolytes, insulin, vitamins, and micronutrients was ensured. In addition, the control group received talcum powder treatment at zusanli and shenque acupoints twice a day, while the treatment group was administered Sini Decoction at zusanli and shenque acupoints twice a day. The Sini Decoction consisted of 6 g of light aconite, 6 g of dried ginger, and 10 g of scorched licorice, which were provided by the Department of Pharmacy at Hangzhou Third People’s Hospital.

### 2.3. Outcome assessments

The demographic data, encompassing age and gender, were meticulously documented. The acute physiology and chronic health evaluation II (APACHE II) scores, sequential organ failure assessment (SOFA) scores, and enteral nutrition tolerance scores were systematically collected on days 1 and 7 posttreatment. The serum indexes, including leukocyte, C-reactive protein, and lactate, were assessed on days 1, 3, and 7 posttreatment. Procalcitonin, interleukin-6 (IL-6), and motilin were evaluated on days 1 and 7 after treatment. Additionally, measurements of abdominal circumference, gastric residual volume, and bowel sounds were recorded on days 1, 3, and 7 following treatment.

The abdominal circumference refers to the horizontal measurement of the abdomen at the iliac crest point. When assessing gastric residual volume, it is essential for patients to fast for a minimum of 6 hours. This volume can be determined by ultrasound through calculating the antral cross-sectional area (usCAS) and subsequently applying it in the following formula^[15]^:


$$\begin{array}{l} Gastric\;residual\;volume\left(mL\right)=27.0+14.6\times usCAS-1.28\times age \end{array}$$

The duration of mechanical ventilation (MV) for patients and the length of stay in the ICU were also recorded.

### 2.4. Statistical analysis

SPSS 22.0 was used for data analysis. For all patients, normally distributed measurement data are expressed as mean ± standard deviation, and were compared by Student *t* test. Non-normally distributed measurement data are expressed as median (interquartile ranges) and were compared by the Mann–Whitney *U* test. Categorical variables are presented as percentages and were analyzed using the χ^2^ test. A value of *P* < .05 indicated statistically significant difference of the analyzed data.

## 3. Results

### 3.1. Characteristics and treatment condition of study participants

A total of 75 patients were enrolled in this study. In the treatment group, 38 patients received Sini Decoction at zusanli and shenque acupoints twice a day combined with live bifidobacterium, lactobacillus, and enterococcus powder. In the control group, 37 patients were treated with talcum powder at zusanli and shenque acupoints twice a day combined with live bifidobacterium, lactobacillus, and enterococcus powder. The demographic characteristics, duration of MV, and length of stay in ICU were presented in Table 1. There was no significant difference observed in terms of age and gender distribution between the 2 groups. The treatment group, consisting of 28 men and 10 women, had an average age of 74.72 ± 15.85, while the control group, comprising 23 men and 14 women, had an average age of 74.19 ± 17.58. There was no statistically significant difference in the duration of patients receiving MV between the 2 groups. However, the length of ICU stay between the 2 groups showed a significant statistical difference (*P* < .05).

**Table 1:** Characteristics and treatment condition of study participants.

	Treatment group	Control group	*P* value
Patients, n(%)	39	36	
Age (years), Mean ± SD	74.72 ± 15.85	74.19 ± 17.58	.893
Gender, n(%)			
Male	29 (74.36)	22 (61.11)	.219
Female	10 (25.64)	14 (38.89)	
Treatment condition			
MV duration (hours), Median (Q1–Q3)	134 (100, 357)	139 (100.75, 376.5)	.682
The length of ICU stay (days), Mean ± SD	9.54 ± 3.98	13.14 ± 5.03	.001

### 3.2. Clinical parameters between the 2 groups

**Figure 1.**
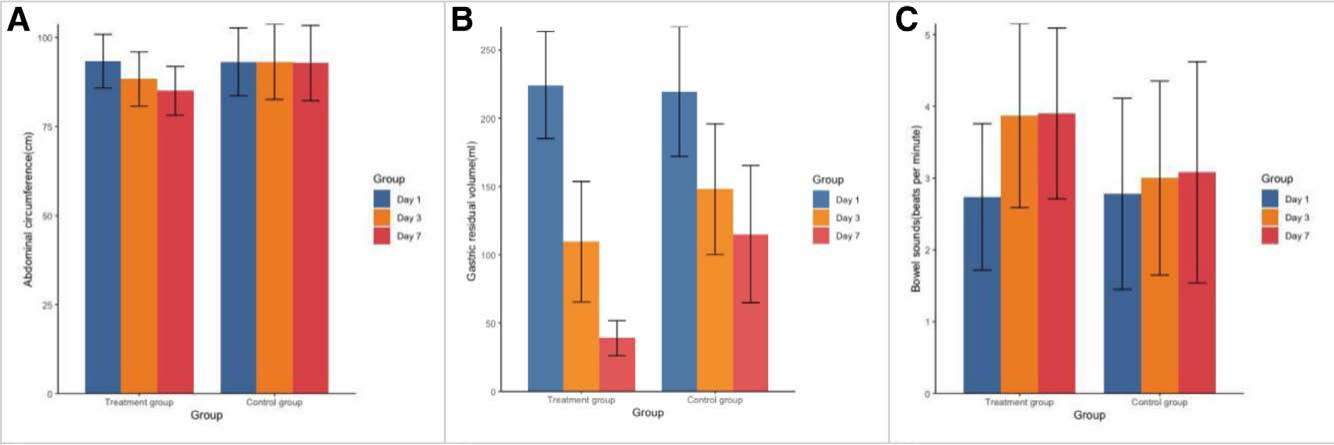
Comparison of the abdominal circumference (A), gastric residual volume (B), and bowel sounds (C) between the 2 groups on days 1, 3 and 7 after treatment.

There is no statistically significant difference in APACHE II scores and SOFA scores between the 2 groups on days 1 and 7 after treatment (Table 2). The results of enteral nutrition tolerance scores indicate that there is no significant difference between the 2 groups on day 1, while a statistical difference was observed on day 7 (*P* < .05). The changes in abdominal circumference were compared among the patients on days 1, 3, and 7 after treatment. Positive results were observed on day 3 and day 7 posttreatment, with *P* values of .025 and .000 respectively. On day 3 after treatment, the abdominal circumference in the treatment group (88.33 ± 7.64 cm) was shorter than that in the control group (93.17 ± 10.57 cm). Similarly, on day 7 after treatment, the abdominal circumference in the treatment group (85.03 ± 6.84 cm) was also shorter than that in the control group (92.83 ± 7.47 cm). The gastric residual volume did not differ significantly between the treatment group and the control group prior to enrollment (*P* = .635). However, at 2 observation points, namely 3 and 7 days after treatment, there was a statistically significant difference between the 2 groups with *P* values of .001 and .000, respectively. When comparing the changes in bowel sounds before and after treatment between the 2 groups, we observed that the treatment group exhibited significantly increased bowel activity compared to the control group on day 3 and 7 posttreatment, with corresponding *P* values of .005 and .012, respectively (Fig. 1).

**Table 2:** Clinical parameters between the 2 groups.

	Treatment group	Control group	*P* value
APACHE II scores			
Day 1, Mean ± SD	18.23 ± 8.02	17.11 ± 7.82	.543
Day 7, Mean ± SD	13.72 ± 7.36	12.61 ± 7.38	.518
SOFA scores			
Day 1, Mean ± SD	5.77 ± 3.55	5.42 ± 3.981	.686
Day 7, Mean ± SD	2.87 ± 2.353	2.14 ± 2.25	.179
Enteral nutrition tolerance scores			
Day 1, Mean ± SD	3.13 ± 2.34	3.58 ± 2.12	.381
Day 7, Mean ± SD	0.90 ± 1.14	1.72 ± 1.52	.009
Abdominal circumference (cm)			
Day 1, Mean ± SD	93.33 ± 7.52	93.15 ± 9.53	.928
Day 3, Mean ± SD	88.33 ± 7.64	93.17 ± 10.57	.025
Day 7, Mean ± SD	85.03 ± 6.84	92.83 ± 7.47	.000
Gastric residual volume (mL)			
Day 1, Mean ± SD	224.23 ± 39.25	219.44 ± 47.49	.635
Day 3, Mean ± SD	109.49 ± 44.01	148.06 ± 47.92	.001
Day 7, Mean ± SD	38.97 ± 12.73	115.08 ± 50.23	.000
Bowel sounds (beats per minute)			
Day 1, Mean ± SD	2.74 ± 1.02	2.78 ± 1.33	.901
Day 3, Mean ± SD	3.87 ± 1.28	3.00 ± 1.35	.005
Day 7, Mean ± SD	3.90 ± 1.19	3.08 ± 1.54	.012

### 3.3. Difference of serum indexes between the 2 groups

The assessment of serum inflammatory indexes includes leukocyte, C-reactive protein, and procalcitonin. We initially compared the levels of serum leukocyte and C-reactive protein on days 1, 3, and 7 posttreatment, but found no significant differences between the 2 groups. Additionally, we compared procalcitonin levels on days 1 and 7 after treatment and concluded that there was no statistically significant difference. The lactate levels in both groups were not significantly different prior to enrollment (*P* = .730). However, there was a significant statistical difference observed on days 3 and 7 after treatment (*P* < .05). The expression of IL-6, an essential factor produced by the innate immune system during the initial response to infection, was compared between 2 groups on days 1 and 7 after treatment. On day 1, no significant difference was observed (*P* > .05), whereas on day 7, a significant increase was noted (*P* < .05). Furthermore, the IL-6 level in the treatment group (4.44 ± 0.96 pg/mL) was found to be lower than that in the control group (6.66 ± 4.11 pg/mL). The motilin levels on the 7th day after treatment were found to be statistically different among various treatment regimens (*P* = .000). Notably, the motilin level in the treatment group was significantly higher than that in the control group (refer to Table 3 for detailed results). Importantly, no significant adverse events were observed during the course of treatment in either group.

**Table 3:** Serum indexes between the 2 groups.

	Treatment group	Control group	*P* value
Leukocyte (×10^9^/L)			
Day 1, Mean ± SD	11.87 ± 5.91	10.00 ± 6.92	.211
Day 3, Mean ± SD	10.22 ± 5.86	8.89 ± 6.03	.335
Day 7, Mean ± SD	9.46 ± 5.04	8.59 ± 3.66	.406
C-reactive protein (mg/L)			
Day 1, Mean ± SD	111.52 ± 99.46	77.60 ± 84.46	.117
Day 3, Mean ± SD	79.14 ± 69.17	52.91 ± 54.30	.073
Day 7, Mean ± SD	52.51 ± 65.54	37.07 ± 50.29	.262
Lactate(mmol/L)			
Day 1, Mean ± SD	1.86 ± 2.01	2.04 ± 2.49	.730
Day 3, Mean ± SD	1.18 ± 0.731	1.85 ± 1.24	.005
Day 7, Mean ± SD	1.01 ± 0.42	1.45 ± 0.76	.002
Procalcitonin (ng/mL)			
Day 1, Mean ± SD	2.81 ± 8.19	4.19 ± 10.26	.524
Day 7, Mean ± SD	0.46 ± 0.71	0.46 ± 0.57	.990
IL-6 (pg/mL)			
Day 1, Mean ± SD	11.21 ± 6.28	13.54 ± 5.97	.105
Day 7, Mean ± SD	4.44 ± 0.96	6.66 ± 4.11	.003
Motilin (pg/mL)			
Day 1, Mean ± SD	58.31 ± 16.74	58.38 ± 23.86	.988
Day 7, Mean ± SD	100.46 ± 30.62	73.64 ± 27.54	.000

## 4. Discussion

When the body experiences sepsis, a gradual series of physiological and pathological reactions occur that pose a serious threat to human health due to its high morbidity and mortality rates. Gastrointestinal dysfunction is a typical manifestation in sepsis patients, and destruction of the gastrointestinal mucosal barrier can cause bacterial translocation, exacerbating sepsis and leading to multiple organ dysfunction syndrome.^[16]^ Therefore, the protection and promotion of gastrointestinal function play a pivotal role in sepsis treatment. TCM can effectively alleviate sepsis-induced gastrointestinal dysfunction due to its potent heat-clearing and detoxifying properties.^[13,17]^ In this study, sepsis patients with gastrointestinal dysfunction were treated using Sini Decoction/talcum powder at acupoints combined with live bifidobacterium, lactobacillus, and enterococcus powder. We collected clinical characteristics and laboratory indicators to assess the treatment outcomes. Sini Decoction is a representative prescription for restoring Yang and overcoming adversity, which can enhance blood circulation and improve gastrointestinal dysfunction in sepsis.^[18–20]^

When sepsis leads to gastrointestinal dysfunction, it results in weakened gastrointestinal activity, increased gastric residual volume, diminished or absent bowel sounds, and an enlarged abdominal circumference due to the accumulation of gastrointestinal gas. Our study revealed that the treatment group exhibited significantly reduced abdominal circumference and gastric residual volume compared to the control group after treatment. Additionally, the bowel sounds in the treatment group were more active than those in the control group, with statistical significance. These findings suggest that Sini Decoction effectively enhances gastrointestinal function by promoting blood circulation and eliminating blood stasis in sepsis patients.^[19,21]^ In TCM, the pathogenesis of sepsis-related gastrointestinal dysfunction is attributed to insufficient vital energy, external pathogenic factors, impaired circulation of Qi-blood in meridians, and internal obstruction caused by toxin heat, stasis blood, phlegm, and dampness. These factors lead to vessel obstruction and damage to the stomach meridian resulting in gastrointestinal dysfunction. TCM considers that pathogen invasion is the primary etiology of gastrointestinal dysfunction in sepsis. Sini Decoction, comprising aconite, dried ginger, and fried licorice, exerts its therapeutic efficacy by dispersing cold and invigorating Yang to rectify inversion. The regulation of disrupted immune condition, reduction in inflammatory responses, reversal of shock, and improvement in microcirculation can enhance the survival outcomes of sepsis patients. It has been hypothesized that quercetin and kaempferol, the core components of Sini Decoction, may modulate HMOX1 expression (a co-expressed gene in Sini Decoction) through associated signaling pathways to target CX3CR1 (a key gene influencing sepsis prognosis) in patients. This modulation could effectively regulate the immune-inflammatory microenvironment of sepsis and mitigate inflammatory responses. Moreover, Gu Y, et al have discovered that CX3CR1 is capable of regulating chemotactic signaling pathways and facilitating the interaction between immune cells and their cytokine ligands and receptors, thereby exerting a protective effect against sepsis.^[19]^

Patients with sepsis are susceptible to gut microbiota dysbiosis. Simultaneously, mounting evidence indicates that the disruption of gut microbiota predisposes individuals to sepsis and exerts a detrimental impact on the outcome of sepsis.^[22]^ The gut microbiome appears to be a promising therapeutic target in sepsis due to its diverse responses to the condition.^[23]^ In our study, all patients received treatment with a live combined powder of bifidobacterium, lactobacillus, and enteroccus. This intervention demonstrated a positive impact on alleviating gastrointestinal dysfunction in sepsis patients. The provision of appropriate nutritional support is crucial in the management of sepsis. We offer comprehensive nutritional support to all patients under study in accordance with the guidelines, encompassing caloric intake, trace elements, vitamins, and other essential components. The provision of nutritional support in sepsis patients is advantageous for maintaining the body’s physiological function, particularly during the hypermetabolic state induced by the disease, thereby preserving negative nitrogen balance in individuals with heightened metabolism. Additionally, nutritional support plays a pivotal role in addressing the imbalance of inflammatory response and reducing the incidence of organ dysfunction among sepsis patients by mitigating inflammation and regulating immune function. The amino acid glutamine, for instance, can provide nutritional support to the liver and serve as a metabolic intermediate to supply cellular energy, thereby mitigating peroxidation damage inflicted on liver cells by oxygen free radicals. Firouzi S, et al have demonstrated that Vitamin C could potentially mitigate excess inflammation, oxidative stress, and regulate cytokine production, which can improve the overall immune response. Zn is also a potent antioxidant and it can prevent the production of free radicals. Zn supplementation may enhance the production of interferon alpha and decrease the production of tumor necrosis factor.^[8]^

The APACHE II scores have been widely used in the ICU setting for accurate estimation of disease severity and mortality prediction.^[24]^ Additionally, the SOFA scores can effectively predict organ dysfunction severity, with a positive correlation observed between APACHE II scores, SOFA scores, and disease severity as well as risk of death.^[25]^ The analysis reveals no statistically significant differences in APACHE II scores and SOFA scores between the 2 groups. However, it is worth noting that the treatment group exhibited a notable reduction in ICU stay duration compared to the control group, suggesting a potential beneficial effect of Sini Decoction on patients with sepsis-related gastrointestinal dysfunction. Leukocyte, C-reactive protein, and procalcitonin can serve as indicators of infection severity. Our study demonstrates that there are no statistically significant differences in these indicators between the 2 groups, suggesting that Sini Decoction has minimal impact on infection control. Therefore, effective management of infections relies on fundamental treatments such as anti-infection therapy and organ function support. The elevation of lactate, a crucial marker for tissue perfusion, is commonly observed in septic patients and is closely associated with organ dysfunction and increased mortality rates. Persistently abnormal lactate levels exceeding 5 mmol/L or higher are indicative of an unfavorable prognosis.^[26,27]^ In our study, a statistically significant difference was observed in the reduction of lactate levels between the 2 groups. On one hand, this may be attributed to effective infection control and improved tissue perfusion. On the other hand, Sini Decoction demonstrated efficacy in improving gastrointestinal ischemia and hypoxia, thereby enhancing gastrointestinal function.

The severity of sepsis is closely associated with IL-6, which not only plays a role in regulating inflammation, but also influences the release of other inflammatory mediators.^[28,29]^ Our study results demonstrated that treatment with Sini Decoction acupoint application led to a more significant improvement in IL-6 levels compared to the control group, indicating its effective potential in ameliorating gastrointestinal dysfunction caused by sepsis-induced inflammation. Pharmacological studies have demonstrated the presence of glycyrrhizin, glycyrrhetinic acid, glycyrrhizin polysaccharide, and other constituents in scorched licorice, which possess potent anti-inflammatory and anti-allergic properties. Additionally, zingerenone and ether extract derived from dried ginger exhibit remarkable anti-inflammatory effects.^[30]^ Motilin levels may also be altered in sepsis patients as a result of systemic inflammatory responses. These alterations can contribute to gastrointestinal dysfunction, such as impaired or absent gastrointestinal peristalsis, thereby further impacting the patient’s nutrient absorption and digestive function.^[31–33]^ We observed a statistically significant difference in motilin levels between the 2 groups before and after treatment. The application of Sini Decoction on specific acupoints may directly target the gastrointestinal tract through meridian conduction, thereby regulating motilin secretion and improving gastrointestinal function.

In conclusion, our present study has observed the application of Sini Decoction at acupoints for the treatment of gastrointestinal dysfunction in sepsis patients. Our findings indicate that acupoint application of Sini Decoction can effectively reduce inflammatory response, improve gastrointestinal function, and shorten ICU stay duration. Although our investigation is considered preliminary, it sheds new light on the management of gastrointestinal dysfunction in sepsis patients. However, it should be noted that the small sample size in our study may introduce bias and therefore further investigations with larger sample sizes and treatment interventions are warranted.

## Acknowledgments

We wish to thank our colleagues in the Department of Medicine for their cooperation with this study.

## Author contributions

**Data curation:** Yuteng Du, Jingjing Hu, Pingping Zhang, Ting'ai Ge.

**Formal analysis:** Jingjing Hu, Pingping Zhang.

**Investigation:** Ting'ai Ge.

**Writing – original draft:** Yuteng Du, Jingjing Hu.

**Writing – review & editing:** Ting'ai Ge, Yidan Zhou.
